# Progenitor species hold untapped diversity for potential climate-responsive traits for use in wheat breeding and crop improvement

**DOI:** 10.1038/s41437-022-00527-z

**Published:** 2022-04-05

**Authors:** Fiona J. Leigh, Tally I. C. Wright, Richard A. Horsnell, Sarah Dyer, Alison R. Bentley

**Affiliations:** 1 The John Bingham Laboratory, NIAB, 93 Lawrence Weaver Road, Cambridge, CB3 0LE UK; 2 Present Address: European Molecular Biology Laboratory, European Bioinformatics Institute (EMBL-EBI), Wellcome Genome Campus, Hinxton, Cambridge, CB10 1SD UK; 3 Present Address: International Maize and Wheat Improvement Center (CIMMYT), Texcoco, Mexico

**Keywords:** Natural variation in plants, Plant hybridization

## Abstract

Climate change will have numerous impacts on crop production worldwide necessitating a broadening of the germplasm base required to source and incorporate novel traits. Major variation exists in crop progenitor species for seasonal adaptation, photosynthetic characteristics, and root system architecture. Wheat is crucial for securing future food and nutrition security and its evolutionary history and progenitor diversity offer opportunities to mine favourable functional variation in the primary gene pool. Here we provide a review of the status of characterisation of wheat progenitor variation and the potential to use this knowledge to inform the use of variation in other cereal crops. Although significant knowledge of progenitor variation has been generated, we make recommendations for further work required to systematically characterise underlying genetics and physiological mechanisms and propose steps for effective use in breeding. This will enable targeted exploitation of useful variation, supported by the growing portfolio of genomics and accelerated breeding approaches. The knowledge and approaches generated are also likely to be useful across wider crop improvement.

## Introduction

Modern crop breeding involving targeted crossing and selection has led to the development of elite, high yielding cultivars. The genetic components of yield have been improved through constant selection for desirable traits, initially in landraces and early varieties and then through trait driven plant breeding (Fradgley et al. 2019). In wheat, the positive impact of this is exemplified by the introduction of semi-dwarfing genes contributing to large increases in yield potential during the so-called Green Revolution (Borlaug 1968). In addition to genetic improvement, agronomic potential is strongly influenced by the environment. Environmental adaptation, through direct breeding and selection, allows for optimisation of yield within the seasonal constraints of a given region (Worland and Snape 2001), control of biotic stresses including pests and diseases (either via crop management or the deployment of disease resistance genes) and targeting of abiotic response, for example to available water (Reynolds et al. 2007), applied fertiliser (Swarbreck et al. 2019) and other production-limiting stresses. The quest to optimise both genetic potential and environmental response for a range of crop production regions around the world is being enhanced by the array of genetic and bioinformatics tools now available (Adamski et al. 2020).

Climate is the driver of environmental change with an impact for crop production capacity (Rosenzweig et al. 2008). Global climate change creates an urgency for the development of cultivars with enhanced resilience to environmental changes in order to secure future food security.

### Expanding the wheat gene pool

Hexaploid wheat (*Triticum aestivum*) arose through a limited number of hybridisation events between a domesticated form of the tetraploid wild emmer wheat, *Triticum turgidum* ssp *dicoccoides* (AABB) and *Aegilops tauschii* (DD) around 10,000 years ago (McFadden and Sears 1946; Cox 1997; Petersen et al. 2006). An intermediate, hulled hexaploid is proposed by Kerber and Rowland (1974) though this is not supported by the archaeological record (Feldman 2001). As bread wheat spread, the crop became adapted to local conditions through selection and the resulting distinct, locally adapted wheats are known as landraces (Camacho Villa et al. 2005; Jones et al. 2012). Landraces of hexaploid wheat have long been used for wheat improvement and are a reservoir of readily available diversity that can be introduced into breeding programmes with relative ease (Wingen et al. 2014). Domestication and subsequent selection have created bottlenecks, reducing genetic diversity in all cultivated wheat species derived from wild emmer wheat including pasta or durum wheat (*T. turgidum* ssp. *durum*) and bread wheat (Tanksley and McCouch 1997; Lopes et al. 2015). Some of this diversity may be reintroduced to bread wheat by interrogating progenitor species for functional variation in target traits.

Tetraploid (AABB) wild emmer wheat has a modern-day range that spans the western Fertile Crescent, southeastern Turkey, and the mountainous regions of eastern Iraq and western Iran. Tetraploid wheats related to wild emmer include emmer wheat (*T. turgidum* ssp*. dicoccum*), a domesticated tetraploid wheat that was widely cultivated prior to the adoption of hexaploid wheat (Salamini et al. 2002), and durum wheat which is widely cultivated, predominantly within the Mediterranean Rim (Martínez-Moreno et al. 2020). Tetraploid wheats are readily crossable with hexaploid wheat and allelic diversity from tetraploid donors or ‘tetraploid derived alleles’ can be introgressed via direct crossing and backcrossing (Ullah et al. 2018).

The diploid progenitor species *Ae. tauschii* (DD) is part of the large *Aegilops* genus (van Slageren 1994) that includes at least 10 diploid and 12 polyploid species (Matsuoka et al. 2015). Many (up to 14; reviewed by Schneider et al. 2008) *Aegilops* species have been used in wheat crossing programmes although most species in the genus are challenging to introgress due to issues with chromosome pairing. This limitation does not exist with *Ae. tauschii* that is characterised as the specific wheat D-genome donor (Kihara 1944, McFadden and Sears 1946) and it has been frequently used for introgression into hexaploid wheat because there is little inhibition of meiotic chromosome pairing between D-genome chromosomes (Kishii 2019). The distribution of *Ae. tauschii* centres on a region to the south of the Caspian Sea and into Azerbaijan. The species range spreads eastward, to Pakistan and western China, via the Kopet Dag Mountains of Turkmenistan, and westward, to central Syria, via the valleys of southeastern Turkey (van Slageren 1994). Although the genus and specific species have a wide geographical range, the genetic diversity of hexaploid wheat’s D-genome is severely limited because of the small number of polyploidisation events that gave rise to it (Giles and Brown 2006). Collections and populations of *Ae. tauschii* have been used to identify useful genes for specific traits, many of which are disease-related (recently reviewed by Kishii 2019) including resistance genes for foliar pathogens and insect pests (Gaurav et al. 2021).

Whilst direct hexaploid × *Ae. tauschii* crossing has been documented, *Ae. tauschii* is predominantly captured via the creation of synthetic allohexaploids made by chromosome doubling of triploid hybrids from an inter-specific AABB × DD cross (also called synthetic wheats or synthetic hexaploid wheats (SHW); Dreisigacker et al. 2008; Mujeeb-Kazi et al. 2013). These can be used to introduce diversity from either or both the tetraploid or diploid donor. Synthetic wheats have been used for breeding to increase diversity (Dreisigacker et al. 2008; Li et al. 2014a), for adaption (Li et al. 2014a), disease resistance (Ogbonnaya et al. 2008) and yield improvement (Jafarzadeh et al. 2016). The creation of octoploid (AABBDDDD) synthetics has also been reported (Chèvre et al. 1989), as have synthetic amphiploids created using introgressions with other wheat species such as *Ae. crassa, Ae. cylindrica* and *Ae. ventricosa* (Mirzaghaderi et al. 2020). These however have not typically been used for downstream breeding applications due to the complexities of ploidy, recombination and tracking introgression segments.

Ancestral wheat species such as *Triticum urartu* (the AA genome donor of bread wheat) and members of the *Aegilops* tribe including *Ae. speltioides* (a relative of the BB genome donor) offer a wealth of diversity in agronomically important traits such as disease resistance (Rowland and Kerber 1974). Many of these species do not cross readily with bread wheat due to the presence of *Ph1* genes preventing recombination between chromosomes (Sears 1977). Instead, a wheat line carrying a mutant allele of *ph1* may be used to induce bread wheat and ancestor homoeologous recombination (Rey et al. 2017). The resulting lines carry large introgressions and development of high-throughput single-nucleotide polymorphism (SNP)-based marker systems designed to screen wild relative species has facilitated rapid validation and tracking of these introgressions (Przewieslik-Allen et al. 2019; King et al. 2017, 2019). Such marker systems are likely to facilitate enhanced and targeted deployment of diversity from wild relatives in breeding programmes in the future.

Climate change is predicted to increase the frequency and intensity of abiotic stress events and their impacts on wheat productivity (Lopes et al. 2015). Here we review the potential for further detailed interrogation of adaptive and physiological variation in wheat’s progenitor species. Work to date has focussed primarily on biotic stresses but there is evidence to support the usefulness of progenitor species for introducing targeted variation for optimising responses to changing climates. Our review demonstrates that there is a gap in the systematic characterisation of progenitor variation specifically for responses to abiotic stress including seasonal adaptation, physiological response, and root system architecture (RSA). Further understanding the genetic and physiological basis of these responses will support future targeted use of progenitor variation for mobilisation into wheat breeding.

## Progenitor species provide additional variation for flowering time and adaptive response

If heat or drought stress occurs during grain filling, abortion of tillers and/or lower kernel weight reduces wheat yield (reviewed by Fleury et al. 2010 and Ni et al. 2018). The manipulation of flowering time can shift grain production away from risk periods, thereby providing an escape strategy. Research undertaken in both Arabidopsis and agronomically important grasses (maize, rice and wheat) over the past 20 years has revealed that floral transition is controlled by complex overlapping genetic pathways (reviewed by Cockram et al. 2007; Colasanti and Coneva 2009). Wheat is a long-day species in which floral initiation is accelerated by exposure to lengthening days. Although the underlying genetics of flowering are complex (reviewed by Hyles et al. 2020), manipulation of the major vernalisation and photoperiod response genes are widely used in wheat breeding programmes to provide adaption to agroeconomic environments (Bentley et al. 2011).

Adaption in terms of phenology is a powerful tool, particularly in marginal environments. Since the 1990s, 25% of reported global wheat yield improvement has come from wheat grown in marginal environments due to breeding for wide adaption (Lantican et al. 2005). Marginal environments and the necessity to mitigate climate-based yield impacts are likely to become more prominent under a climate change scenario. Climate change is also likely to have impacts on crop production in temperate and cold regions of the world where flowering is a function of both winter cold and spring heat (Yu et al. 2010). Temperate cereals grow across a wide range of semi-arid environments but show marked reductions in productivity (Reynolds et al. 2010) and yield (Lobell and Field 2007) at high temperatures. Increased temperatures in winter may delay fulfilment of vernalisation requirement (a prolonged period of cold, non-freezing, temperatures required for subsequent competence to flower) resulting in later flowering, although increased spring temperatures could mask or offset this (Yu et al. 2010). In areas of high latitude and altitude the effect could be exacerbated, as plants in these regions are particularly sensitive to temperature cues. Vernalisation in wheat is controlled by the major *Vrn-1* locus (Dubcovsky et al. 1998) with the additional *Vrn-2* and *Vrn-3* loci also contributing to variation (Yoshida et al. 2010). Hexaploid wheat has three homoeologous *Vrn-1* loci (denoted *-A1*, *-B1* and *-D1*) located on group 5 chromosomes. Dominant alleles confer a spring growth habit meaning that a cold period is not required for induction of flowering.

Natural plant populations often have wide flowering time variation (Grazzani et al. 2003) and therefore progenitor species offer potential functional genetic variation for fine-tuning adaptive response. In hexaploid wheat, the photoperiod response *Ppd-1* loci are a homoallelic series on group 2 chromosomes (Worland and Snape 2001; Beales et al. 2007; Bentley et al. 2011). In tetraploid (AABB) wheat Wilhelm et al. (2009) described two mutations of the *Ppd-A1* gene leading to photoperiod insensitivity (PI) and early flowering. These effects have also been confirmed in hexaploid and SHW (Bentley et al. 2011). However, screening of ancestral tetraploids (*T. dicoccoides* (*n* = 122) and *T. dicoccum* (*n* = 276)) for these mutant *Ppd-A1a* alleles revealed no variation, suggesting that these are photoperiod sensitive species, and that insensitivity arose post-domestication, being first observed in *T. durum* landrace accessions as well as in collections from southern Europe (Italy, Spain, France), North Africa and North America (Bentley et al. 2011).

Diversity in flowering time has been further characterised by several studies in tetraploids wheats (Nishimura et al. 2018; Wright et al. 2020; Würschum et al. 2019). Alleles of the *Ppd-A1* associated with early flowering (but distinct from the *Ppd-A1a* alleles described by Wilhelm et al. 2009) were detected in emmer wheat by Nishimura et al. (2018) who also identified an early heading date QTL associated with *Vrn-A3*. This QTL was found to be a cis-element GATA box in *Vrn-A3* (located on chromosome 7AS), which suppressed the late-flowering (photoperiod sensitive) *Ppd-A1b* allele (Nishimura et al. 2018). A QTL controlling flowering time was also reported on 7B linked to *Vrn-B3* in an emmer mapping population (Wright et al. 2020). Takenaka and Kawahara (2012) identified novel loss of function alleles in tetraploid *Ppd-A1* in emmer wheat that do not confer PI but may induce small variations in flowering time.

Compared with work in tetraploid progenitors, little is currently known about the diversity of flowering time response in the diploid wheat progenitor *Ae. tauschii*. Matsouka et al. (2008) assessed natural flowering time variation in a collection of 200 accessions representing the latitudinal range (30°N–45°N) of the species. Flowering time phenotypes could be divided into early-, intermediate- and late-flowering groups that enabled detection of geographical patterns: with early-flowering lines being dominant in southern regions compared to late-flowering lines in northern regions. However, the impacts of environmental differences varied between the western and eastern parts of the species range preventing a clear attribution of genetic effects (Matsouka et al. 2008).

Range expansion occurs when species adapt beyond native habitats and has been documented for *Ae. tauschii* associated with shifts in phenology and seed production ability (Matsuoka et al. 2015). Of the species within the *Aegilops* genus, *Ae. tauschii* is the only diploid species to have expanded its range east and Matsuoka et al. (2015) suggest that early flowering at least partially explains range expansion into Asia. Further work by Koyama et al. (2018) used a F_2_-based QTL mapping approach to determine genetic differences between photoperiod sensitive and insensitive lines. This allowed for mapping of a QTL locus on 5DL for heading under short days, proximal to the *Vrn-D1* locus, along with three QTLs (one on 4D, two on 7D) for flowering under field conditions. Quantitative variation for vernalisation was also observed in *Ae. tauschii* accessions (Koyama et al. 2018).

Golovnina et al. (2010) identified spring variants of *Ae. tauschii* including a recessive *Vrn-D1* allele. Vernalisation-insensitive accessions of the species have been previously described in germplasm originating from Pakistan and Afghanistan (Tanaka and Yamashita 1957; Tsunewaki 1966) but there is little evidence for the use of derived alleles in breeding. Takumi et al. (2011) used 211 accessions collected across the *Ae. tauschii* habitat range to assess flowering in the absence of vernalisation. Sequencing of the *Vrn-D1* locus and haplotype analysis revealed distinct variation in *Ae. tauschii*, including a large deletion leading to a loss of vernalisation requirement (Takumi et al. 2011). The authors however conclude that this deletion is discreet from mutations in *Vrn-D1* dominant alleles in hexaploid wheat, indicating that the loss of vernalisation requirement in the progenitor and domesticated forms of wheat occurred separately, but followed a similar mutational event (Takumi et al. 2011). Understanding the vernalisation response and the interactions between *Vrn-1* and other genes (e.g., the floral repressors *Vrn-2*), particularly at high temperatures will be important for future resilience breeding.

Dixon et al. (2019) demonstrate that diverse material can provide variants of many of these genes and that understanding their interactions can potentially facilitate their use for incorporating resilience to temperature fluctuations. Overall, although significant variation has been reported for adaptive response in wheat progenitor species, gaps exist in deployment into breeding. We propose that this is due to two main factors: the lack of resolution available for genetic trait dissection in wild progenitors and the confounding effects of genotype × environment. Many of the alleles or QTLs described from progenitor species to date have not been genetically resolved and many co-locate in forward genetic studies. The availability of sequenced progenitor collections (e.g., Gaurav et al. 2021) is likely to improve the resolution of novel alleles from progenitors, thereby enabling their rapid extraction and validation. This will also likely address the other current limitation in separating the confounding effects of environment and masking effects of interacting loci. Overlapping flowering time pathways introduce functional redundancy, particularly in hexaploid wheat, and they are influenced by multiple environmental factors. Therefore, the priority requirement for extraction of useful functional adaptive trait variation from progenitors is rapid and accurate assaying, extraction and validation of variants to enable quantification of phenotypic effects independent of genetic background and environmental effects.

## Novel physiological traits can potentially be mined from progenitor species

Cultivars bred for high yield potential under optimal conditions typically maintain performance in moderately stressful environments (Richards et al. 2002; Foulkes and Reynolds 2015; Voss-Fels et al. 2019). The yield potential of a crop can be simplified to a function of light interception (LI), harvest index (HI) and radiation use efficiency (RUE, Reynolds et al. 2009). Progression in crop breeding has brought HI and LI close to theoretical maximum (Long et al. 2006) indicating that selection for improved RUE may be the most rewarding opportunity for breeders to increase yield potential. RUE is effectively the slope of correlation between dry matter content at harvest and total intercepted radiation (Murchie et al. 2009). Optimising RUE is key to utilising available resources when breeding for variable or resource-limited environments. Thus, enhancing crop canopy photosynthesis is an important breeding target and progenitor species may offer novel physiological variation that can be exploited in breeding.

**Fig. 1:**
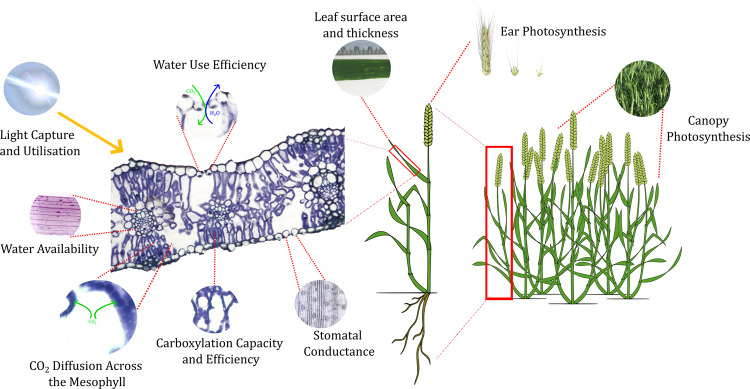
A schematic showing key targets for photosynthetic improvement where diversity from wild relatives could be utilised to increase productivity or stress tolerance in modern wheat. The flag leaf cross-section highlights important traits underpinning CO_2_ assimilation on a standardised leaf area basis. When considering photosynthesis on a plant or canopy basis, other targets for improvement include organ size, ear photosynthesis and CO_2_ assimilation across the whole canopy.

Crop photosynthesis is a complex process, consisting of dynamic networks from the molecular to canopy level (Fig. 1). When considering CO_2_ assimilation expressed on a standardised leaf area basis (*A*), there are numerous morphological and biochemical traits underpinning performance. Past experiments have highlighted that wheat progenitors harbour higher *A* than hexaploid wheat cultivars (Evans and Dunstone 1970; Austin et al. 1982). Since domestication, due to selection programmes for other agronomic traits, there has been limited historic selection pressure from breeders on leaf photosynthetic capacity (Driever et al. 2014). If progenitor diversity can be captured to target a single aspect of the process of photosynthesis giving a moderate increase in flag leaf *A* of modern wheat then, as the canopy carbon fixation is an integrated process multiplied over the entire growing season there could be consequential overall improvements in RUE and yield (Parry et al. 2011). To harness diversity from wild relatives, components driving high *A* need to be identified to facilitate their use in targeted genetic dissection, direct use in pre-breeding and future application in wheat breeding using marker- and phenotypic-based screening methods.

The determinants of *A* (Fig. 1), and thus potential targets for improvement, include components that govern the rate of delivery of CO_2_ to the sites of carboxylation; the availability of products from photochemical reactions; and downstream enzyme-regulated mechanisms of the Calvin–Benson cycle. Within these components, superior characteristics found in progenitors can be targeted to improve either photosynthetic productivity or tolerance under environmental stress in modern cultivars (e.g., Merchuk-Ovnat et al. 2016a, b).

The delivery of CO_2_ to the sites of carboxylation is governed by several diffusive boundaries, particularly those imposed by the leaf stomata. When stomata are closed, water loss is minimal, but the closed pores act as the sole limitation to carbon fixation (Farquhar et al. 1982). Therefore, there is a fundamental trade-off between the flux of CO_2_ entering the leaf and flux of H_2_O exiting (Lawson and Blatt 2014). The proportion of CO_2_ gained in relation to H_2_O transpired is termed instantaneous water use efficiency (WUE) (Farquhar and Richards 1984). Wheat progenitors have been shown to maintain higher instantaneous WUE in drought-prone conditions compared to hexaploid wheat (Li et al. 2017). Furthermore, Merchuk-Ovnat et al. (2016a) found that introgressions from *T. dicocciodes* into hexaploid wheat were linked to greater grain yield under drought. Plants originating from drier climates, such as wild relatives, would require increased hydraulic supply to the leaves to maintain photosynthesis under increased evaporative loss (Scoffoni et al. 2016). Austin et al. (1982) found higher stomatal and vein densities in tetraploid wheat flag leaves compared to hexaploid varieties, which could reflect a strategy for maintaining *A* in drought-prone environments. An alternative strategy could aim to reduce stomal density to minimise water loss and improve drought tolerance (Hughes et al. 2017). As variation in leaf stomatal density and size has been observed across wheat ploidy levels (Dunstone et al. 1973; Khazaei et al. 2009), wild relatives could be a genetic reserve for optimising the balance between CO_2_ and water loss depending on the targeted breeding environment.

In rice, a distinct group of landraces known as aus-type rice (McNally et al. 2009) evolved and were cultivated under environmental stress conditions in India and Bangladesh. Aus-type rice has been shown to be a valuable source of novel diversity; varieties developed from this material have been shown to be highly tolerant of drought (Henry et al. 2011) and heat stress (Li et al. 2015). A range of physiologies underpins such adaptation including increased rooting depth and lateral root formation resulting in increased water uptake, thus reduced canopy temperature prevented stomatal closure and prolonged photosynthetic activity in the drought-tolerant rice lines (Henry et al. 2011). In combination, heat and drought stress have a negative additive effect on many aspects of wheat plant physiology (reviewed by Tricker et al. 2018), and identifying a suite of tolerance traits pertaining to a fine balance of gas exchange, WUE and assimilation from wild relatives is a breeding target in order to maintain yield under combined stresses.

Another diffusive boundary that acts as a limitation to *A* is the diffusion of CO_2_ across the mesophyll (*g*_*m*_, Fig. 1). This boundary is governed by mesophyll anatomical or biochemical features (Evans et al. 2009; Flexas et al. 2012). There has been limited investigation of how *g*_*m*_ varies across wheat ploidy levels. The grasses are generally considered to have comparatively high *g*_*m*_ (Flexas et al. 2012), which may have decreased through the domestication process, as negative correlations have been observed with *g*_*m*_ and potentially desirable traits such as leaf mass area (Gu et al. 2012). Mesophyll cell size is thought to have increased across wheat ploidy, with ancestral species possessing smaller cells (Dunstone and Evans 1974; Wilson et al. 2021). Smaller mesophyll cells may facilitate higher *g*_*m*_ due to an increased surface area for gas exchange (Lundgren and Fleming 2020). Further work is required to establish if the comparatively high rates of *A* found within progenitor species are driven by higher *g*_*m*_.

Improved photochemistry is another trait targeted for improvement (Fig. 1). In a large wheat wild relative comparison using 41 species, McAusland et al. (2020) identified accessions that outperformed modern varieties in traits linked to photochemistry, including *T. dicoccoides* and lines from the *Amblyopyrum* and *Aegilops* genera that demonstrated high Photosystem II (PSII) operating efficiency or electron transport. They hypothesised that high rates of maximum electron transport and carboxylation resulted in high photosynthetic capacity in some wild relative accessions. An introgression from wild emmer into bread wheat has also been linked to improved electron transport rate during booting (Merchuk-Ovnat et al. 2016b). Under high light intensity, not all captured energy is utilised in photochemistry and plants have developed mechanisms for dissipating possibly detrimental excess energy through photoprotection (Demmig-Adams and Adams 1992). This protection process is termed nonphotochemical quenching (NPQ). When the leaf is returned to lower light intensities, the time required for the relaxation of NPQ is a limit to crop productivity (Kromdijk et al. 2016). Improved RUE and photosynthetic efficiency have been achieved through manipulation of the NPQ process through genetic engineering (see: Kromdijk et al. 2016; Hubbart et al. 2018). However, while there has been promising diversity observed in NPQ kinetics in diverse relatives of wheat (McAusland et al. 2020), the degree to which natural variation could be exploited from wild ancestors still needs to be determined.

The determinants, and limitations, imposed at the sites of carboxylation relate to the enzyme-regulated mechanisms of the Calvin–Benson cycle (Fig. 1). Johnson et al. (1987) concluded that a higher capacity for mesophyll photosynthesis may be linked to variation in CO_2_ assimilation across wheat ploidy. Demand for CO_2_ is restricted by the carboxylation and oxygenation activities of the enzyme Rubisco (Farquhar et al. 1980), the capacity and efficiency of this enzyme is a major bottleneck in raising wheat yields (Parry et al. 2011). Prins et al. (2016) demonstrated the superior Rubisco catalytic properties of several wheat genotypes (including progenitors) compared to the modern wheat variety Cadenza when assessed across different temperatures. Scafaro et al. (2012) found a wild relative of rice maintained a higher activation state of Rubisco under higher temperatures compared to domesticated rice, which was linked to the high heat tolerance of the wild relative. Progenitors of wheat, originating from warmer climates, may also possess superior Rubisco kinetics, which could be utilised in breeding for marginal environments; this requires further systematic characterisation. Rubisco is also responsible for catalysing the oxygenation of ribulose 1,5-bisphosphate. Photorespiration is the energetically expensive process of converting the by-products of the oxygenation reaction and is a significant constraint on wheat productivity (Long et al. 2006; Parry et al. 2011).

In tobacco, South et al. (2019) showed that genetic engineering of pathways linked to photorespiration produced promising improvements to biomass production and photosynthetic efficiency. Yield penalties linked to photorespiration may lesson under future predicted climates but will still remain important (Walker et al. 2016). While the most promising gains in improving photorespiration losses may be through genetic engineering routes, targeting superior Rubisco characteristics from relatives could hold promise, such as selection for the Rubisco specificity factor that may be higher in plants from drier environments (Galmés et al. 2005). Natural sources of variation in Rubisco kinetics (e.g., Prins et al. 2016) may be a more readily available tool for breeders to utilise in ongoing selection programmes compared to genetic engineering routes.

Targeting photosynthetic improvement should also be considered on both a leaf and canopy basis (Fig. 1). When considering CO_2_ capture on a per leaf basis, the total organ surface area and thickness are key components. The smaller leaf area typical of progenitor species (Evans and Dunstone 1970) may have more concentrated photosynthetic capacity (Long et al. 2006). McAusland et al. (2020) found that the thicker and narrower leaves found in wild relatives underpinned a higher maximum carboxylation rate, which was also supported by the observed negative relationship between specific leaf area and photosynthetic capacity. Furthermore, leaf surface area and *A* are typically negatively correlated (Evans and Dunstone 1970; Austin et al. 1982) and a major challenge in utilising photosynthetic diversity from wild relatives will be transferring high *A* found in progenitor lines into a larger flag leaf typical of modern wheat. This could be addressed by using existing pre-breeding material (derived from progenitors) to screen for genotypes that show a deviation from the negative correlation between leaf area and *A*. As a first step, segregating pre-breeding material could be used to extract extreme individuals based on leaf area and used either for reciprocal recurrent selection or for bulk segregant analysis to move forwards the genetic understanding of the link between *A* and leaf area.

Long et al. (2006) outlined that the efficiency of the canopy to intercept light is controlled by canopy characteristics linked to size, architecture, longevity, speed of development and closure. Successful breeding efforts across recent decades have limited opportunities for improvements in canopy LI efficiency (Zhu et al. 2010). Furthermore, canopy architecture has been optimised through domestication (Li et al. 2014b), suggesting wild ancestors may not be a useful source of variation. Canopy conditions are very heterogeneous, particularly in terms of light distribution (Horton 2000). A crop canopy that responds quickly to these changes will be more efficient in maximising resource capture (Taylor and Long 2017). Fast photosynthetic and photoprotection induction has been observed in wild rice accessions (Acevedo‐Siaca et al. 2021) and in wild wheat relatives (McAusland et al. 2020), respectively. Incorporating these faster light transition responses into modern wheat could be an objective for improving resource capture. Targeting earlier photosynthetic improvement before canopy closure is another potential route for improvement, as pre-anthesis photosynthesis is known to correlate with grain yield (Gaju et al. 2016; Carmo-Silva et al. 2017). Gaju et al. (2016) found at a pre-anthesis growth stage (during the onset of stem extension) a synthetic-derived hexaploid genotype maintained higher *A* than its recurrent hexaploid parent.

Taken together, there is good evidence to support the need for further characterisation of the component traits underpinning photosynthesis in wheat progenitors. Although much of the trait variation described is likely to be quantitatively controlled, there is an opportunity to identify specific progenitor accessions for direct use as donors in physiological pre-breeding. In addition, the development of protocols and tools for rapid screening of these physiological traits will enhance future genetic dissection. At present most methods require detailed experimentation and specialist equipment so the development of predictive phenotyping tools also offers promise to enable accurate forward genetic studies to discover trait-linked markers, and the selection of favourable variants in marker-assisted breeding. This is likely to yield significant benefits for breeding offering new potential to transfer higher WUE for drought tolerance or increased *A* from progenitor species.

Another strand of potential variation for further investigation towards application is the photosynthetic potential of reproductive tissues in progenitor species (Fig. 1). Ear photosynthesis is heritable, varies across different wheat genotypes and is an important determinant of grain yield (Molero and Reynolds 2020), highlighting the importance of ear photosynthesis as a breeding target. Li et al. (2017) found that ears of *T. dicoccoides* maintained higher CO_2_ assimilation during grain-filling when compared to hexaploid wheat, along with higher WUE under drought stress. Progenitor wheat species, particularly tetraploids, typically have a larger awn surface area than hexaploid wheat (Blum 1985). As a photosynthetic organ, awns have been reported to have high instantaneous WUE (Blum 1985; Weyhrich et al. 1995) potentially explaining why in a drought-prone environment, the presence of awns is reported to be beneficial to grain yield (Evans et al. 1972). Other components of the ear may also harbour useful stress tolerance characteristics, Araus et al. (1993) found that WUE was 33% higher in the ear bracts compared to the leaf blade using carbon isotope analysis, linking the higher efficiency to a lower *g*_*s*_ and the xeromorphic features of the ear bracts (glumes, paleas and lemnas). Under heat stress, positive correlations have been observed between grain yield and the contribution of ear photosynthesis to grain yield (Molero and Reynolds 2020). Progenitors originating from drier and hotter environments may possess strategies, such as high ear CO_2_ fixation linked to the preservation of photosynthesis under unfavourable conditions and these could become increasingly useful for adapting modern wheat to more marginal environments.

Although little data exist on the quantitative differences in ear photosynthesis in wheat progenitors, and their relative contributions under stress, further work is warranted. As breeders seek to incorporate additional diversity into their programmes, the selection of progenitor donors with high ear CO_2_ fixation could be prioritised. Further evidence is required to confirm the consistency of photosynthetic contributions from the presence of awns. If consistently higher photosynthetic capacity can be demonstrated without reducing photosynthetic activity in other parts of the plant, then this trait can be readily incorporated as a breeding target due to the additional benefit and ease of phenotypic and genotypic selection. In many regions, awned wheat varieties predominate making it likely this benefit is already present and fixed, but it could also be applied where awned varieties are not widespread, and/or to prioritise selections within segregating pre-breeding material derived from progenitors.

## Progenitor species are a source of new root system architecture ideotypes

RSA plays a pivotal role in drought tolerance and nutrient acquisition and enhancing root systems is a target for improving climate resilience (recently reviewed by Ober et al. 2021). Deeper roots can extract more water from subsoils, particularly during late developmental stages and grain fill, thereby improving yield in water limiting environments (Manschadi et al. 2010). However, the characterisation of mature RSA in wheat can be time consuming making it difficult to use as a selection target in breeding (Richard et al. 2015). Techniques that use early rooting traits (seminal root angle and seminal root number, e.g., the clear pot system developed by Richard et al. 2015) or root crowns extracted from the field at maturity (e.g., using the shovelomics method adapted from maize (Trachsel et al. 2011)) can be used to infer wheat RSA (Fradgley et al. 2020). A ‘pasta strainer’ technique described by El Hassouni et al. (2018) allows characterisation of the mature root system when grown within a perforated basket submerged in the field. All these tools allow RSA of genotypes to be characterised into wide or narrow/deep rooting types.

Wheat progenitor species may be used to augment the diversity in RSA that exists in the bread wheat gene pool. Tetraploid wheats have been shown to offer RSA diversity; using recombinant inbred lines of durum × wild emmer, QTLs for drought resistance and related traits were mapped (Peleg et al. 2009). Marker-assisted selection (MAS) enabled the QTL regions to be introgressed into both durum and hexaploid wheat (Merchuk-Ovnat et al. 2016a; b). This produced one hexaploid wheat isogenic line with introgression of a QTL from chromosome 7A of the wild emmer donor showing greater productivity (biomass, flag leaf area and grain yield) and photosynthetic capacity than the recurrent parent when grown under water limiting conditions. RSA was found to differ in this line, with greater development of deep roots and associated root tips whilst under drought stress (Merchuk-Ovnat et al. 2016a). This RSA enhanced the plant’s ability to access water at a greater soil depth and conferred greater drought tolerance as subsoil water levels are generally more stable than those in the upper layers of the soil.

Iannucci et al. (2017) identified 17 QTLs relating to root and shoot morphology in a durum × emmer wheat population, three of which were previously undescribed (two for the number of root tips and one for rooting depth). Root morphology QTL co-segregated with the height reducing *Rht-B1* gene on chromosome 4B, indicating these alleles are involved in the control of both root and shoot traits, with tall plants having longer and larger root systems in this population. However, Christopher et al. (2013) found no co-segregation of root traits with dwarfing genes and most studies agree that root and shoot development are under the control of different sets of loci (Iannucci et al. 2017). QTL clusters for root morphology traits have also been reported to coincide with those for thousand grain weight and yield (Maccaferri et al. 2008; Iannucci et al. 2017) but further work is required to resolve these interactions. El Hassouni et al. (2018) found that in trials with low water availability, durum accessions with deep roots achieved a 37–38% yield increase but suffered a 20–40% yield penalty in irrigated environments.

Previous work has shown yield and biomass increases in synthetic-derived wheat lines can be attributed to a greater proportion of deep roots (Reynolds et al. 2007). Becker et al. (2016) also demonstrated that increased rooting depth and fine root mass allowed for the maintenance of plant growth under drought stress in two synthetic wheat lines, thus maintaining yields. However, a third synthetic line lacked deep roots but tolerated drought stress through increased stomatal density and reduced stomatal aperture (Becker et al. 2016). Recently Liu et al. (2020) detected eight QTL associated with drought tolerance in a SHW × commercial wheat F_2_ population with most of the positive alleles attributable to the *Ae. tauschii* (four QTLs) or tetraploid (durum; two QTLs) components of the synthetic. Ober et al. (2021) reviewed the range of wheat trait variation reported in wheat as well as summarised available evidence linking deeper roots to access to soil moisture.

Understanding the RSA diversity available in the wheat gene pool will allow the selection of targeted root types to suit environmental conditions such as drought or waterlogging, and nutrient availability. This remains a medium- to long-term breeding objective as there is still relatively little known about the heritability, environmental and management independence of RSA in elite cultivars (Fradgley et al. 2020). As highlighted by Ober et al. (2021) many upstream research questions remain including the mechanisms by which architectural traits impact water and nutrient acquisition. In addition, there remains a gap in understanding the linkage and direction of interactions between root and agronomic/crop production traits, and their environmental dependencies. Progenitor species typically have a wide eco-geographical adaption range, and it is proposed that this is likely to confer functional RSA variation. Whilst surveying large collections of progenitors for RSA variation is possible, more rapid progress is likely through the identification of pre-breeding material (capturing progenitor variation) with contrasting root types and comprehensive analysis of the linkages between trait variation and root functions. As for photosynthetic traits, high-throughput screening methods that can be scaled and applied for forward genetic screens and MAS are likely to accelerate progress in exploiting progenitor variation for RSA.

## Prospects for climate-responsive breeding across crop species

Major and minor crops worldwide are likely to face both new limitations and opportunities for maintaining and increasing productivity due to changing climates. The identification of useful variation as described for wheat progenitor species and the successful application of approaches to mobilise it into cultivated wheat can serve as an exemplar for other crops. In addition to supporting productivity, this will also incentivise the search for useful variation in their progenitors and wild relatives. Exploration of progenitors or crop wild relatives has already begun in a variety of crop species (e.g., legumes (Porch et al. 2013; Coyne et al. 2020) apples (Volk et al. 2015) and numerous others (reviewed in (Hajjar and Hodgkin 2007; Dempewolf et al. 2017)) to identify genomic regions linked to phenotypes of interest for both biotic and abiotic stresses. For major cereals such as rice and barley, there are already examples of the successful introgression of traits linked to climate change adaptions such as drought tolerance (Talame et al. 2004; Zhang et al. 2006) and flowering traits (Ishimaru et al. 2010; Wiegmann et al. 2019). For minor cereal grain crops there are few confirmed examples to date, e.g., sorghum (reviewed in (Ananda et al. 2020)), pearl millet (reviewed in (Sharma et al. 2020)), finger millet (blast resistance (Akech et al. 2016)), oats (reviewed in (Ociepa 2019)) and rye (plant height and yield (Falke et al. 2009)), indicating that allocating resources to the exploration of diversity within progenitors and wild relatives would reveal further useful adaptations that could improve the resilience of these crops to changing climates. Examples of monocot crops, their progenitor species and breeding priorities linked to changing climates are shown in Table 1.

**Table 1:** Monocot crops and their progenitor species or wild relatives that offer genetic diversity for targeted crop improvement.

Crop	Progenitors	Breeding priorities linked to climate stresses	References
Maize (*Zea mays*)	Teosinte (*Z. mays* ssp. *parviglumis*)	Drought, heat, waterlogging	Mano and Omori 2013; Challinor et al. 2016
Rice (*Oryza sativa*)	*O. rufipogon*	Drought, heat, flooding, salinity, C4 photosynthesis	Zhang et al. 2006; Ishimaru et al. 2010; Covshoff and Hibberd 2012; Singh et al. 2021
Wheat (*Triticum aestivum*)	*T. turgidum* ssp *dicoccoides* and *Aegilops tauschii*	Drought, heat, C4 photosynthesis	Covshoff and Hibberd 2012; Lopes et al. 2015
Barley (*Hordeum vulgare*)	*H. vulgare* ssp. *spontaneum*	Drought, heat, waterlogging, C4 photosynthesis	Setter and Waters 2003; Talame et al. 2004; Covshoff and Hibberd 2012; Weigmann et al.^ 2019^
Sorghum (*Sorghum bicolor*)	*S. bicolor* subsp. *verticilliflorum*	Cold, drought, heat	Ananda et al. 2020
Pearl millet (*Pennisetum glaucum*)	*P. glaucum* subsp. *monodii*	Drought and heat	Sharma et al. 2020
Oats (*Avena sativa*)	*A. ventricosa, A. longiglumis, A. insularis, A. canariensis* and *A. agadiriana*	Cold, drought and heat, C4 photosynthesis	Covshoff and Hibberd 2012; Ociepa 2019
Rye (*Secale cereale*)	*S. cereale* subsp*. vavilovii*	Drought and heat, C4 photosynthesis	Covshoff and Hibberd 2012; Miedaner and Laidig 2019
Finger millet (*Eleusine coracana)*	*E. coracana* subsp. *africana*.	Drought and salinity	Mirza and Marla 2019

Opportunities also exist to transfer desirable characteristics from minor to major cereal grain crops. An avenue that holds much promise, along with numerous technical challenges, is the incorporation of the C_4_ photosynthetic pathway, a characteristic of a C_4_ crop (e.g., sorghum or millet), into a C_3_ crop (e.g., rice or wheat). The C_4_ pathway utilises a carbon concentrating mechanism to diminish photorespiration, a process that takes place at the sites of carboxylation that limits productivity in C_3_ crops. The C_4_ pathway evolved due to increased abiotic stress, including heat and drought, which are conditions that can enhance photorespiration (Sage 2004). There is scope for breeding photosynthetic improvements within C_4_ crop species (von Caemmerer and Furbank 2016). However, major cereal crops are still cultivated in climates that favour photorespiration, meaning the enhanced water and nitrogen use efficiency characteristics of the C_4_ pathway is an attractive breeding target for C_3_ crops (Mitchell and Sheehy 2006). Climate change could exacerbate this need further, which has contributed to a concerted effort to incorporate the C_4_ pathway into C_3_ crops, in particular rice (e.g., www.c4rice.com). Challenges still need to be overcome before these improvements are available to the breeding community and C_3_ wild progenitors may provide a more accessible source of improvement for major C_3_ crops.

## Opportunities exist to use genomics to accelerate the use of progenitors in crop breeding

Whilst traditional breeding approaches have been successfully used to cross cultivated materials with their wild relatives to introduce traits of interest, the success rate varies between species and becomes increasingly difficult with more distantly related species. There also remain barriers to using genomics-based advances to accelerate the uptake of novel alleles. Linkage drag is traditionally one of the major barriers to incorporating diversity from progenitors. Here, unwanted genes are introgressed simultaneously with a targeted region from a donor into the desired background. Backcross breeding is typically used to increase the recurrent parent (background) genotype and reduce unwanted genes. This strategy can be complemented by MAS, allowing the selection of a specific trait based on a linked genetic marker. MAS can be employed to facilitate more accurate introgression from a progenitor donor and reduce linkage drag from a wild background (Tanksley et al. 1989). This has been used successfully to make introgressions from several wild relatives into domesticated wheat (Nevo and Chen 2010; Merchuk-Ovnat et al. 2016a; King et al. 2017). Beyond linkage drag, other factors can pose issues to capturing wild diversity. The merging of genomes across wheat species can lead to intergenomic gene suppression (Feldman and Levy 2012). This phenomenon leads to the silencing of homoeologous genes and is reported to be common in hexaploid bread wheat (Bottley et al. 2006). This poses a potential problem for utilising newly synthesised wheats in pre-breeding programmes. However, the establishment of homoeologs does not necessarily result in functional silencing or suppression through dominance; phenotypes can be influenced by an additive dosage effect or complex interactions linked to the homoeologs (Borrill et al. 2015). Another potential roadblock is the genomic instability and radical changes which can occur because of allopolyploidization (Kraitshtein et al. 2010). However, there is evidence to suggest the severity of these changes may be of little consequence to the overall development of the plant (Zhao et al. 2011). Recent advancements in next-generation sequencing provide an opportunity for increasing our understanding of the functional genomics that underpin relationships across homoeologs (reviewed in Borrill et al. 2015). These tools could contribute to providing an improved understanding of the functional genetics of newly formed pre-breeding resources such as synthetic wheats incorporating progenitor diversity.

Advancements in sequencing technologies have facilitated the discovery of large numbers of DNA markers in crop species. In wheat (Winfield et al. 2012), this has led to the development of numerous genotyping platforms (Adamski et al. 2020) that have aided the application of QTL mapping and have enhanced the accessibility of diversity in progenitors and related species (Winfield et al. 2016, 2018; Wingen et al. 2017). SNPs are very effective markers in high-throughput genotyping due to their abundance across the wheat genome (Rimbert et al. 2018). Specific platforms have been developed to characterise wheat progenitors and wild relatives, including the Axiom^®^ HD Wheat Genotyping Array (Winfield et al. 2016) and the Axiom^®^ Wheat-Relative Genotyping Array (Przewieslik-Allen et al. 2019) in addition to arrays developed for elite varieties (e.g., Axiom^®^ Wheat Breeder’s Genotyping Array; Allen et al. 2017). The wheat-relative array has been used to aid the introgression of the diploid wheat-relative *Ambylopyrum muticum* into a hexaploid wheat background through MAS (King et al. 2017). Furthermore, the Wheat Breeders’ array has been used in several studies for identifying QTLs in tetraploid wheat (Lucas et al. 2017; Wright et al. 2020). Low-cost genotyping platforms designed to demonstrate potential genetic variability between progenitor species and elite varieties are a tool of growing importance in exploring and harnessing diversity and have been deployed in many crops such as barley (Bayer et al. 2017), rice (Chen et al. 2013) and maize (Xu et al. 2017).

The availability of sequenced genomes from crop species, for example, the annotated reference genome assembly of the wheat cultivar Chinese Spring (International Wheat Genome Sequencing Consortium et al. 2018) augmented by the multiple genome assembly of Walkowiak et al. (2020) improve our understanding of the size and context of targeted introgressions through knowledge of the physical chromosome location of markers used for selection. In addition, the resources can improve our understanding of synteny with ancestral genomes (Grewal et al. 2018). Introgression fragments can be queried to identify the genes and any potentially favourable alleles present (Cheng et al. 2019). Due to the reducing expense of sequencing technologies (Jia et al. 2018), the number of cultivars sequenced is increasing, including many important elite wheat varieties (e.g., the 10+ genomes project: www.10wheatgenomes.com; Montenegro et al. 2017; Walkowiak et al. 2020). Increasing the number of modern wheat varieties sequenced, or genotyped through high-density marker arrays, will help characterise the haplotype diversity within the modern wheat gene pool. Haplotypes present in low diversity may reflect regions that have been under past selection (Fradgley et al. 2019) or where variation has been lost due to the domestication bottleneck (Haudry et al. 2007). Regardless, using this knowledge, targeted comparisons can then be made with extended progenitor gene pools to capture novel haplotypes (Uauy 2017). This comparison is being accelerated in wheat by the availability of increasing numbers of progenitors sequenced, including *Ae. tauschii* (Luo et al. 2017), *T. urartu* (Ling et al. 2013), *T. dicoccoides* (Avni et al. 2017) and *T. durum* (Maccaferri et al. 2019). A recent study by Cheng et al. (2019) compared re-sequenced genome data from a mixture of cultivated and progenitor wheat accessions, flagging regions of past introgression and identifying haplotype blocks that are nearly completely fixed in cultivated varieties. These regions of low diversity highlight the potential for identifying regions to target for improving genetic diversity from progenitor species.

In addition to the characterisation of haplotype diversity, enhanced sequencing resources will also support genetic mapping, cloning and functional characterisation from progenitor species. Kishii (2019) summarised the progress in generating genetic and physical mapping resources for *Ae. tauschii* documenting the progression from the early use of restriction fragment length polymorphism mapping in *Ae. tauschii* mapping populations (Gill et al. 1991) through to single sequence repeat genotyping (Nishijima et al. 2018). This supported the production of a 10 K *Ae. tauschii* Infinium SNP array by Luo et al. (2013) and the draft sequence of *Ae. tauschii* (Luo et al. 2017). The availability of reference genomes supports the use of data-driven approaches to selections, including linking phenotype to gene expression as demonstrated by Gálvez et al. (2019) for drought tolerance. This highlights the potential impact of understanding gene networks underpinning traits, and how genomics may identify novel breeding targets (Gálvez et al. 2019).

Resources supporting reverse genetics have also been developed in progenitor species with Targeting Induced Local Lesions in Genomes populations available in the wheat tetraploid (durum wheat Kronos; Krasileva et al. 2017) and diploid species (*Ae*. *tauschii*; Rawat et al. 2018), as well as being available for hexaploid wheat (cultivar Cadenza; Krasileva et al. 2017). A wheat exome capture was developed to focus sequencing efforts on exons, thereby reducing sequencing costs (Winfield et al. 2012). Along with genome sequences, these provide a useful resource for allele mining and gene discovery and could be used in future to support gene identification and cloning directly from the progenitor species. Direct cloning of favourable genes from progenitor species has been demonstrated using a combination of association genetics and resistance gene enrichment and sequencing (AgRenSeq; Arora et al. 2019). This method has been used to both discover and clone functional stem rust resistance genes in a panel of diverse *Ae. tauschii* accessions (Arora et al. 2019). Molecular breeding technologies provide the potential to directly introduce useful variation discovered in one crop into another, either by the introduction of the gene via genetic transformation or gene editing to introduce variation within homoeologous genes. The efficiency of the approaches discussed in this review remains to be seen for different genes and crops and can be impacted by the genetic background of particular varieties, but identifying a set of variants that already exist in nature and that can be used to introduce variation within genes of interest is an exciting prospect for the future.

## Summary

There is a wealth of variation present in crop progenitor species for traits of relevance to plant breeding including flowering time, physiological response and RSA. Although initial characterisation demonstrates that functional variation exists, there remains a significant opportunity to systematically characterise this variation in order to make it accessible for use in breeding. In particular, more work is required to fully understand the genetic and physiological basis of progenitor trait variation in order to accurately inform future breeding strategies. The growing availability of sequencing and genomics tools offers great potential for targeted and accelerated progress in the systematic use of functional progenitor variation. The advances in use of wheat progenitors and the techniques developed for the capture of novel diversity may be applicable for the improvement of other cereal crop species.
